# Single and Repetitive Surge Reliability of 1200 V Asymmetric Trench SiC MOSFETs Under Various Gate Biases

**DOI:** 10.3390/mi17070823

**Published:** 2026-07-10

**Authors:** Menglin Yan, Zhizhe Wang, Dazheng Chen, Yuncong Li, Yongle Zhong, Yuansheng Li, Jun Luo, Hao Xia

**Affiliations:** 1 State Key Laboratory of Wide-Bandgap Semiconductor Devices and Integrated Technology, Xidian University, Xi’an 710071, China; 24181214135@stu.xidian.edu.cn; 2 State Key Laboratory of Reliability Technology for Electronic Components, China Electronic Product Reliability and Environmental Testing Research Institute, Guangzhou 511370, China; liyuncong@m.scnu.edu.cn (Y.L.); 19584569324@163.com (Y.Z.); liyuansheng@ceprei.com (Y.L.); luojun@ceprei.com (J.L.); xiahao@ceprei.com (H.X.); 3 Guangzhou Wide Bandgap Semiconductor Innovation Center, Guangzhou Institute of Technology, Xidian University, Guangzhou 510555, China; 4 The School of Electronic Science and Engineering (School of Microelectronics), South China Normal University, Foshan 528225, China; 5 The School of Materials Science and Engineering, South China University of Technology, Guangzhou 510641, China

**Keywords:** asymmetric SiC MOSFET, body diode, single and repetitive surge stress, different gate voltages, surge capability

## Abstract

The parameter degradation and failure mechanisms of 1200 V asymmetric trench-type (AT) silicon carbide (SiC) metal oxide semiconductor field-effect transistors (MOSFETs) under various single and repetitive surge currents, with various gate bias voltages (V_GS_) of 0 V, −5 V, and −10 V, are systematically investigated in this work. It is indicated that V_GS_ has no impact on the single surge reliability, with the same maximum single surge current (SSC_max_) under different V_GS_. However, during repetitive surge stress (90% and 60% SSC_max_), the maximum surge cycles have increased as V_GS_ increases from −10 V to 0 V. It may be caused by the enhancement of channel-assisted leakage conduction, allowing more surge current to flow through the channel. It is concluded from gate capacitance (Cg-Vg) and low-frequency noise (LFN) characterizations that lower V_GS_ increases SiC/SiO_2_ interface defect density, accelerating parameter degradation during single and repetitive surge stress. Both chip and package failures are observed for single and repetitive surge stress. For single surge stress, the device failure has resulted from the melted source Al as the metal erodes and penetrates through the interlayer dielectric and the ohmic contact layer between the source metal and the SiC-doped region, respectively, leading to a three-terminal short circuit. For repetitive surge stress, the device failure has been caused by the penetration of Al metal into the interlayer dielectric, leading to a gate-source short circuit. This comprehensive research provides valuable guidance for enhancing the surge reliability of SiC MOSFETs.

## 1. Introduction

Power devices play a pivotal role in power electronics technology, performing critical functions such as electric energy conversion, power control, and system protection. With the rapid development of new energy technologies such as electric vehicles and other related fields, the performance and reliability of power devices directly influence the advancement and application scope of power electronics technology. In recent years, silicon carbide (SiC) metal oxide semiconductor field-effect transistors (MOSFETs) are increasingly replacing traditional silicon-based devices due to their advantages of high-frequency operation, high voltage capability, superior thermal stability, fast switching, and low power losses, and they have become the preferred choice for high power applications such as new energy vehicles, photovoltaic inverters, and industrial drives [1,2].

Commercially available SiC MOSFETs are currently categorized into three dominant architectures: planar-gate structure, symmetric double-trench structure, and asymmetric-trench (AT) structure [3]. Compared with planar and symmetric trench structures, AT SiC MOSFETs have attracted more and more attention with their advantages of simultaneous optimization of conduction performance and gate oxide reliability. The device utilizes the (11$\bar{2}$0) crystal plane for its conductive channel, which exhibits an extremely low interface state density and oxide trap concentration at the SiC/SiO_2_ interface. This significantly enhances channel electron mobility, thereby substantially reducing the device’s specific on-state resistance (R_DS(on)_). Concurrently, a deep p+ shielding region has been introduced on one side, and it effectively suppresses electric field crowding at the trench corners, preventing premature breakdown of the gate oxide layer [4].

As power density and switching frequency continue to increase, surge reliability has become a critical reliability concern, limiting the large-scale application of SiC MOSFETs [5]. Under actual operating conditions such as motor startup, lightning strikes, and so on, the devices endure surge stresses that far exceed their rated current [6]. Although the deep p+ well optimizes the electric-field distribution for AT SiC MOSFETs, a distorted current distribution readily occurs during freewheeling of the intrinsic body diode, which directly induces gate oxide degradation and a thermal concentration effect, drastically deteriorating the device’s surge ruggedness [7].

Currently, the surge reliability of SiC MOSFETs are the focus of research [8,9,10,11,12,13,14,15,16,17,18]. However, relevant studies have mainly focused on conventional planar-gate structures and symmetrical double-trench structures [8,9,10,11,12,13,14,15,16,17], with relatively limited investigations into asymmetric trench structures. Meanwhile, most existing literature has only studied either single or repetitive surge reliability for SiC MOSFETs [9,10,11,12,13,14,15,16,17,18], and there is still a lack of systematic investigation on the parameter degradation and failure mechanism under both single and repetitive stresses. Furthermore, although the impact of gate-source voltage (V_GS_) amplitude on single-surge reliability has been studied in the literature [11,12,13,14,15,16,17,18], research on the effect of V_GS_ on the repetitive surge reliability of SiC MOSFETs is relatively scarce, especially for AT SiC MOSFETs. In addition, the effect of varying repetitive surge current amplitudes on SiC MOSFET reliability has rarely been studied.

Thus, the parameter degradation and failure mechanisms of 1200 V AT SiC MOSFETs under single and repetitive surge stress with varying V_GS_ and surge current amplitudes are systematically investigated in this work. Three samples are tested for each condition during the surge experiments to ensure the reliability of the results. During the surge test, the degradation behaviors of key parameters, including three-terminal resistance, threshold voltage (V_TH_), on-state resistance, body diode voltage drop (V_SD_), drain-source leakage current (I_DSS_), gate-source leakage current (I_GSS_), subthreshold swing (S.S.), gate capacitance (Cg-Vg), and low-frequency noise (LFN) characteristics, are summarized. Meanwhile, failure modes and mechanisms of the devices are clarified through multiple characterization techniques, such as X-ray inspection, scanning acoustic microscopy (SAM), and post decapsulation scanning electron microscopy (SEM).

## 2. Experiment Introduction

**Figure 1:**
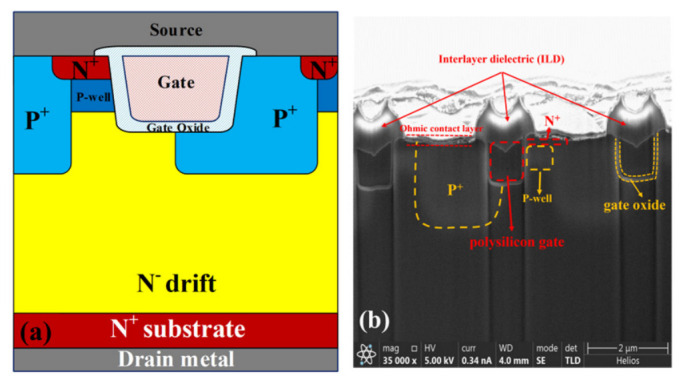
AT-MOSFET: (**a**) structural diagram and (**b**) cross-sectional view.

**Figure 2:**
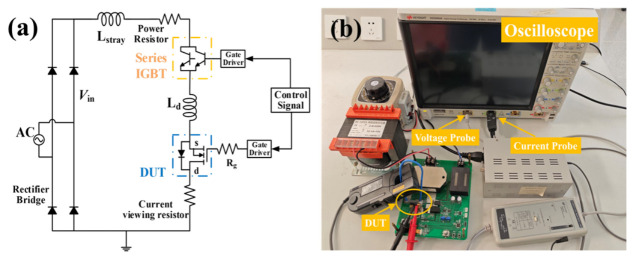
(**a**) Simplified schematic of the surge current test circuit. (**b**) Surge test platform for the SiC MOSFETs.

**Figure 3:**
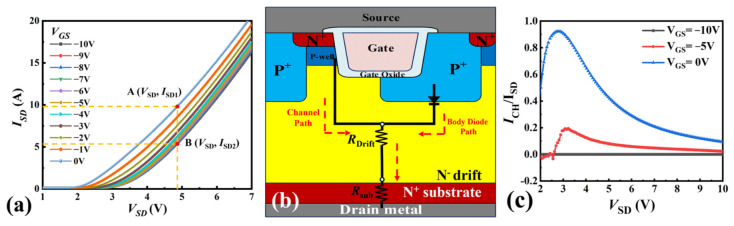
(**a**) Body diode characteristic curves of the DUTs at different gate voltages. (**b**) The surge current path for V_GS_ between −9 V and 0 V. (**c**) Variation in the channel current proportion with V_SD_ under different V_GS_.

The devices under test (DUTs) are commercially available 1200 V AT SiC MOSFETs with a rated current of 36 A. Figure 1 illustrates the device structure and cross-sectional view. During the surge pulse test, half-wave sinusoidal currents of a 10 ms duration are injected through the DUTs. The simplified test circuit and surge test platform are shown in Figure 2a,b, and this circuit is employed to assess the single and repetitive surge pulse current withstanding capabilities and parameter degradation behavior of the SiC MOSFETs. Figure 3a presents the third-quadrant I–V characteristic curves. When V_GS_ is less than or equal to −9 V, the curves remain essentially unchanged, indicating that the current conduction is dominated by the body diode with a negligible contribution of the channel-assisted leakage conduction, resulting in an identical I_SD_ at the same V_SD_. As V_GS_ increases from −9 V to 0 V, the I_SD_ increases under the same V_SD_. At this stage, the MOS channel is partially turned on, providing an additional current path. The surge current path is shown in Figure 3b, where the current flows from the source to the N^−^ drift region through both the body diode and the MOS channel in parallel before reaching the drain [18,19]. Equation (1) defines the proportion of channel current (I_CH_) to the source-drain current I_SD_ [20]:

(1) $$\frac{I_{CH}}{I_{SD1}}=\frac{I_{SD1}-I_{SD2}}{I_{SD1}}.$$

Point A (V_SD_, I_SD1_) is selected on the curve at V_GS_ = 0 V and point B (V_SD_, I_SD2_) is selected at V_GS_ = −10 V, sharing the same voltage coordinate (V_SD_) as point A. The difference between the two points arises from the current at point A (I_SD1_), which includes both the current flowing through the channel (I_CH_) and the body diode, whereas the current at point B (I_SD2_) only contains the current flowing through the body diode. As is visually evident from Figure 3c, with the increase in V_SD_, the proportion of I_CH_ at V_GS_ = 0 V and V_GS_ = −5 V shows a downward trend. In the main range of V_SD_, the body-diode current dominates for a larger fraction, indicating that the surge current continues to be dominated by body diode conduction.

Based on these observations, surge current tests are conducted under three V_GS_: V_GS_ = 0 V, –5 V, and –10 V, corresponding to different channel states, respectively (Table 1). V_TH_ is measured in accordance with the JEDEC standard JEP 183A using the constant current method [21]. To suppress the hysteresis effect in the extracted V_TH_, a positive gate preconditioning pulse V_GS(MAX)_ is applied before the down-sweep phase, and V_TH_ is extracted during the down-sweep at V_GS_ = V_DS_ and I_DS_ = 5 mA.

## 3. Results and Discussion

### 3.1. Single-Pulse Surge Test

**Figure 4:**
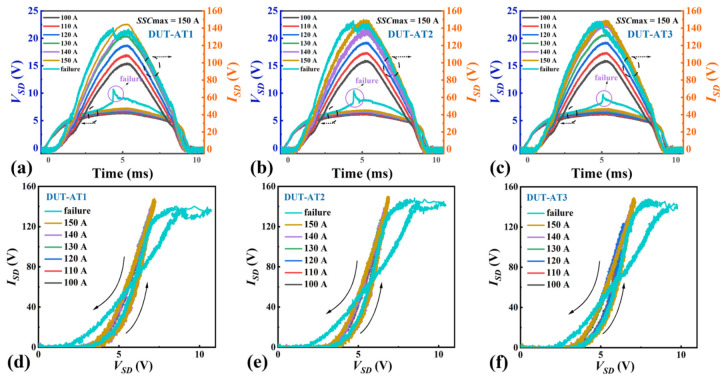
Single surge current waveforms and surge trajectories of the DUTs under different gate voltages: (**a**,**d**) DUT-AT1, (**b**,**e**) DUT-AT2, and (**c**,**f**) DUT-AT3.

Firstly, a single-pulse surge test is conducted on the AT SiC MOSFETs under various V_GS_. The surge current starts at 100 A and is increased in steps of 10 A until device failure occurs. A 10-min interval is maintained between the successive pulses to ensure adequate cooling and prevent thermal accumulation. The surge current waveforms and corresponding current trajectories are shown in Figure 4, where the V_GS_ for DUT-AT1, DUT-AT2, and DUT-AT3 is set at 0 V, −5 V, and −10 V, respectively. A voltage spike is observed in V_SD_ at the moments of device failure. Under the maximum single surge current stress (SSC_max_), the waveforms of V_SD_ and I_SD_ remain consistent. The SSC_max_ for all devices is uniformly 150 A. The surge trajectories of the DUTs exhibit a counterclockwise direction, indicating that the body diode conduction mechanism is dominant under these conditions [22].

Subsequently, a first-order estimation of the junction temperature under surge stress is conducted using the transient thermal impedance method (Foster thermal equivalent circuit) [23]. The instantaneous power dissipation is calculated from the measured voltage and current waveforms as *P*(t) = *V*(t)*I*(t). The single-pulse dissipated energy is calculated as follows:

(2) $$E_{pluse}=\int_{0}^{t_{p}}V_{SD}\left(t\right)I_{SD}\left(t\right)\;dt.$$

The junction temperature rise is then estimated by convolving the power waveform with the transient thermal impedance (*Z*_thjc_(t)) obtained from the device datasheet. The device junction temperature is calculated as follows:

(3) $$T_{j}\left(t\right)=T_{c}+∆T_{jc}\left(t\right)=T_{c}+P\left(t\right)*Z_{thjc}(t),$$

where T_c_ = 303 K is defined as the case temperature. Although this method does not provide spatial temperature distribution as finite-element thermal simulation does, it provides a conservative first-order correlation between the surge current stress and junction temperature rise. The relevant parameters for various V_GS_ biases are listed in Table 2. Although V_GS_ slightly changes the voltage drop and pulse energy, the measured SSC_max_ remains unchanged within the experimental current-step resolution. This indicates that the single surge failure threshold is not sufficiently sensitive to the small V_GS_-induced difference in single-pulse energy. Conversely, repetitive degradation is significantly affected by the V_GS_-induced difference, as detailed in Section 3.2 and Section 3.3.

In this work, the DUTs’ surge capability is defined as:

(4) $$Surge\;capability=\frac{I_{FSM}}{I_{rated}},$$

where I_FSM_ is the DUTs’ surge current at the moments of failure and I_rated_ represents the rated current of the DUTs at 298 K, which is 36 A [19]. After each surge pulse, the resistances between the three terminals of the devices (R_GS_, R_SG_, R_DS_, R_SD_, R_GD_, and R_DG_) are measured for preliminary evaluation. The values are recorded when the devices fail, and these are summarized in Table 3. The relatively low resistances observed across all three terminals under the different V_GS_ indicate that short circuit occurs among the terminals of the DUTs, along with internal structural damage.

**Figure 5:**
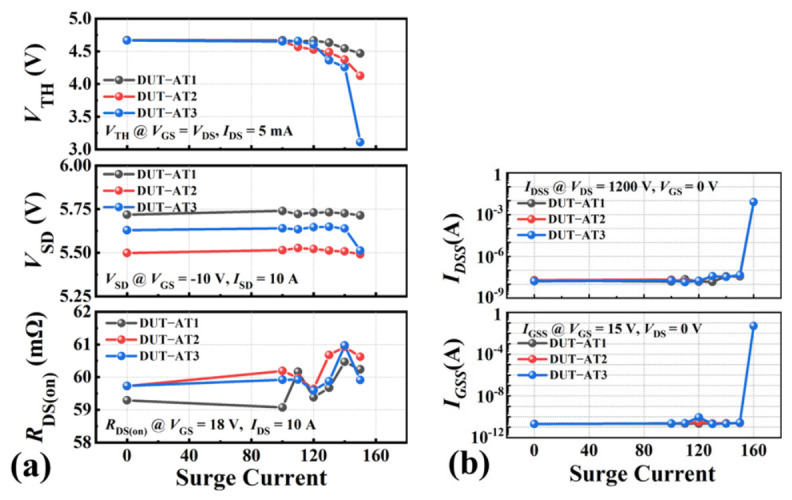
Degradation of electrical parameters for DUT-AT1, AT2, and AT3 at varying V_GS_: (**a**) V_TH_, V_SD_, R_DS(on)_, and (**b**) I_DSS_, I_GSS_.

Figure 5 illustrates the degradation of the electrical parameters after each surge pulse. V_TH_, V_SD_, and R_DS(on)_ become unmeasurable when device failure occurs. V_GS_ significantly influences parameter degradation prior to total failure. DUT-AT1, AT2, and AT3 exhibit negative V_TH_ shifts under varying V_GS_. DUT-AT3 shows the most severe degradation, with a 33.34% reduction in V_TH_, while DUT-AT1 and AT2 show decreases of 4.36% and 11.61%, respectively. The more pronounced V_TH_ negative shift at a more negative V_GS_ is attributed to the stronger electric field at the gate–oxide interface of the DUTs (from the substrate to the gate electrode), which elevates the impact ionization rate along the sloped trench sidewalls and ultimately facilitates the injection of holes into the gate oxide layer [24]. Simultaneously, R_DS(on)_ shows a slight upward trend following the increasing surge current. The ∆V_SD_ remains below 1% for DUT-AT1 and AT2, yet AT3 exhibits the largest V_SD_ change, with a reduction of approximately 2.1%. Drain-source leakage current (I_DSS_) and gate leakage current (I_GSS_) remain nearly identical under different V_GS_ until device failure, where both currents sharply increase to the milliampere (mA) level, indicating the loss of gate control over the channel and voltage blocking capability between the drain and source.

**Figure 6:**
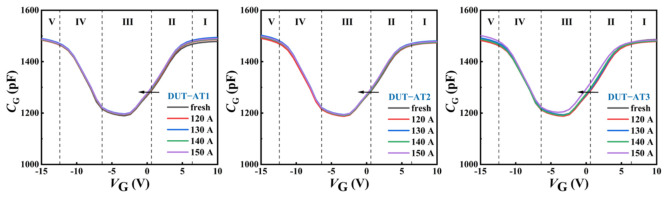
Cg-Vg characteristics for DUT-AT1, AT2, and AT3.

The changes in the Cg-Vg characteristics of the DUTs are presented in Figure 6. The waveform changes primarily occur in Region II and Region III, indicating that the degradation is concentrated at the edge of the gate oxide layer or the trench sidewall of the devices [25,26]. As gate-bias voltage stress decreases from 0 V to −10 V, the Cg-Vg curves shift further to the left, consistent with the V_TH_ degradation trend. It is indicated that the increased density of the interface traps caused by more negative V_GS_ intensifies gate-oxide deterioration and correspondingly lowers V_TH_ under single-pulse surge current stress, consistent with the literature [27].

**Figure 7:**
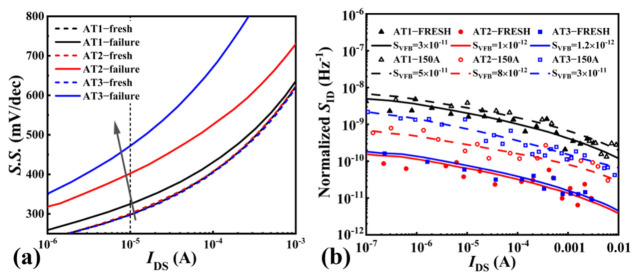
Degradation of (**a**) subthreshold swing and (**b**) normalized noise power spectral density for DUT-AT1, AT2, and AT3.

Figure 7a shows the variation in S.S. under different V_GS_. S.S. increases significantly after surge stress, indicating degradation of the gate oxide layer. Under the effect of surge stress, additional electron traps are generated at the SiC/SiO_2_ interface, with higher trap density observed at lower V_GS_. Figure 7b illustrates the relationship between the normalized noise power spectral density measured at 10 Hz and I_DS_ before and after the single surge current stress. The low-frequency noise power spectral density of DUT-AT1, AT2, and AT3 increase by 0.67, 7, and 10.83 times, respectively, after single surge stress compared to their initial values, which is consistent with Cg-Vg and V_TH_ variations. This may be attributed to the higher localized electric field stress caused by the more negative V_GS_, which amplifies the generation of interface traps and oxide traps under surge stress. These traps facilitate the carrier trapping and release processes [28,29].

### 3.2. Repetitive Surge Test at 90% SSC_max_

For the repetitive surge test, the time interval between each surge pulse is set to 10 s to avoid heat accumulation within the devices. The values of 90% and 60% of SSC_max_ are selected for the repetitive surge current stress. Here, 90% SSC_max_ serves as the extreme condition for assessing thermomechanical failure margins, while 60% SSC_max_ acts as the moderate condition for reflecting gradual degradation. These stress levels effectively accelerate the degradation process while avoiding sudden the catastrophic failures caused by a single-pulse surge, consistent with existing studies where surge amplitudes typically fall within 60–90% of SSC_max_ [8,9,10,30].

V_GS_ for DUT-AT4, AT5, and AT6 are set at 0 V, −5 V, and −10 V, respectively, with a repetitive surge current amplitude of 135 A, corresponding to 90% of the maximum single surge current. The maximum surge cycles to failure and the three-terminal resistances at the point of failure are summarized in Table 4. It is indicated that the number of surge current cycles the device can withstand decreases as V_GS_ becomes more negative. This may be caused by a weaker contribution of channel-assisted leakage conduction, which leads to more current flowing through the body diode during repetitive surge current stress. Meanwhile, DUT-AT4, AT5, and AT6 exhibit a significant reduction in gate-source resistance when the devices fail, indicating that gate-source short circuit occurs.

**Figure 8:**
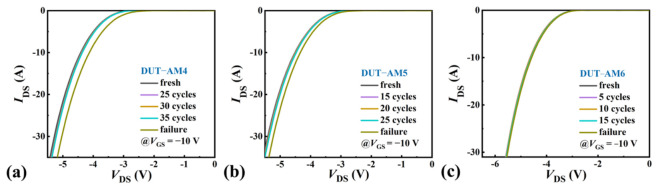
Degradation in the third-quadrant characteristics of the DUTs under repetitive surge current stress of 90% SSC_max_: (**a**) DUT-AT4, (**b**) DUT-AT5, and (**c**) DUT-AT6.

Figure 8 shows the changes in the third-quadrant characteristics of DUT-AT4, AT5, and AT6 under repetitive surge stress. When the devices fail, the knee voltage and forward drop of the body diode in DUT-AT4 decrease from 2.9 V and 4.3 V to 2.3 V and 4.1 V, respectively, indicating a gate-source short circuit and reduction in the body diode conduction barrier. However, for DUT-AT5, the knee voltage and forward voltage drop of the body diode show smaller changes compared to DUT-AT4. For DUT-AT6, the V_SD_ near 2.9 V remains unchanged from its initial value, indicating that the device maintains reliable blocking capability.

**Figure 9:**
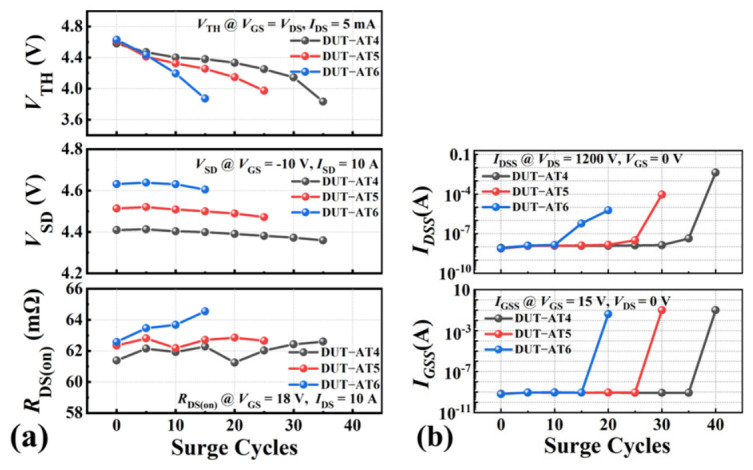
Degradation of electrical parameters for DUT-AT4, AT5, and AT6 at varying V_GS_: (**a**) V_TH_, V_SD_, R_DS(on)_ and (**b**) I_DSS_, I_GSS_.

The degradation trends of the electrical parameters with the surge cycles are shown in Figure 9. Prior to complete failure, DUT-AT4, AT5, and AT6 exhibit significant degradation in V_TH_ as the surge cycles increase, where the degradation is fastest at V_GS_ = −10 V, consistent with the single surge stress result. R_DS(on)_ increases by 1.9%, 0.5%, and 5.16% for AT4, AT5, and AT6, respectively, while the changes in V_SD_ remain below 1%. I_GSS_ of DUT-AT4, AT5, and AT6 escalates to the mA range when the devices fail, confirming the inevitable damage to the gate oxide layer. However, only DUT-AT4’s I_DSS_ reaches the mA level, losing its blocking capability.

**Figure 10:**
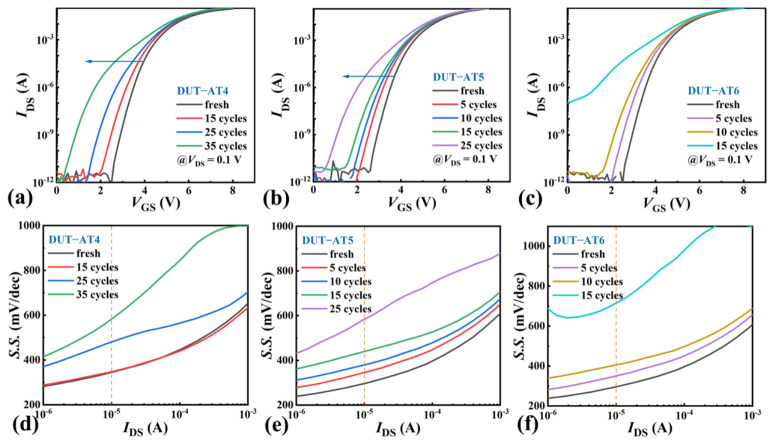
Degradation of the transfer curves and subthreshold swing for the DUTs during the repetitive surge current test: (**a**,**d**) DUT-AT4, (**b**,**e**) DUT-AT5, and (**c**,**f**) DUT-AT6.

**Figure 11:**
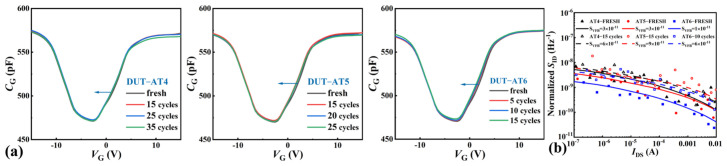
(**a**) Cg-Vg characteristics for DUT-AT4, AT5, and AT6. (**b**) Degradation of the normalized noise power spectral density for DUT-AT4, AT5, and AT6.

In Figure 10, the transfer characteristics of the DUTs shift to the left with the increasing surge stress cycles; meanwhile, the S.S. increases as the surge cycles increase. The degradation trend of DUT-AT6 is the most pronounced, demonstrating that V_GS_ significantly influences the stability of the gate–oxide interface. Figure 11a illustrates the degradation trends of the Cg-Vg characteristics of the DUTs. As the applied V_GS_ becomes more negative, the negative shift in the Cg-Vg curves within the 0 V to 5 V range of Vg intensifies, indicating more trap generation in the gate oxide layer, consistent with the results of V_TH_. Figure 11b reveals that the low-frequency noise power spectral density of DUT-AT4, AT5, and AT6 after surge stress is 2, 3, and 6 times greater than the initial values, respectively, consistent with the Cg-Vg results. It is shown that the larger negative bias may amplify the generation of defects, consistent with the results for the single surge test.

### 3.3. Repetitive Surge Test at 60% SSC_max_

**Figure 12:**
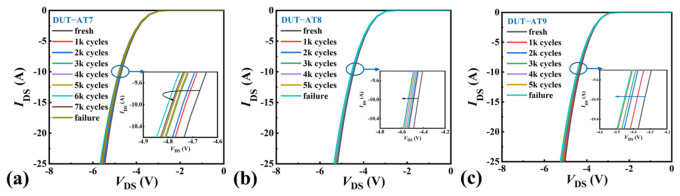
Degradation in the third-quadrant characteristics of the DUTs under repetitive surge current stress of 60% SSC_max_: (**a**) DUT-AT7, (**b**) DUT-AT8, and (**c**) DUT-AT9.

V_GS_ for DUT-AT7, AT8, and AT9 are set at 0 V, −5 V, and −10 V, respectively, with a repetitive surge current of 90 A, corresponding to 60% of the maximum single surge current. The maximum surge cycles to failure and three-terminal resistances when the DUTs fail are presented in Table 5. The surge endurance of DUT-AT7, AT8, and AT9 is in the range of thousands of cycles, which is hundreds of times that for 90% SSC_max_. This may be caused by the decrease in the surge current amplitude. The resistances between the gate and source terminals of the devices decrease to the kilohm (kΩ) range, indicating the presence of a leakage path between the gate and source, which is consistent with the 90% SSC_max_ test results. For DUT-AT7, the resistances between the drain-gate and source-drain terminals decrease to the tens of megohms (MΩ) range, yet the device still retains its blocking capability. Unlike the case of the large surge current stress, the variation trends of the third-quadrant characteristics under small surge current stress show no significant dependence on V_GS_, as shown in Figure 12. The knee voltage and forward voltage drop of the body diode exhibit a gradually degrading trend.

**Figure 13:**
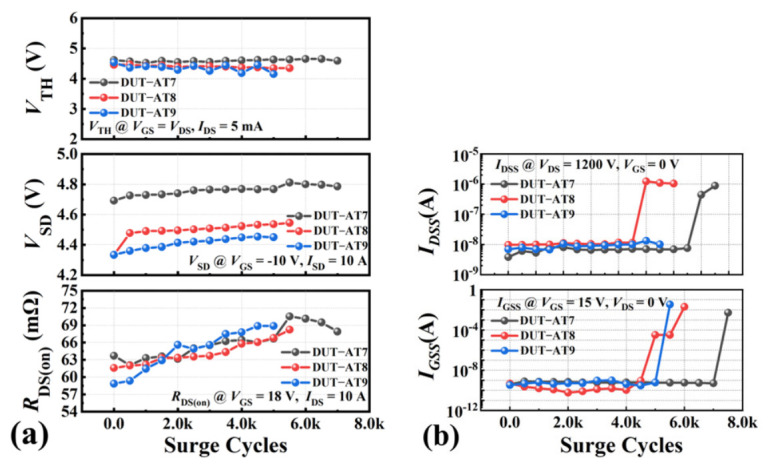
Degradation of electrical parameters for DUT-AT7, AT8, and AT9: (**a**) V_TH_, V_SD_, R_DS(on)_ and (**b**) I_DSS_, I_GSS_.

As shown in Figure 13, after the devices withstand small surge current stress, DUT-AT7, AT8, and AT9 have failed after 7000, 5500, and 5000 surge cycles, respectively. The degradation in V_TH_ is relatively slow, while the changes in R_DS(on)_ and V_SD_ are more pronounced compared with those for 90% SSC_max_. The I_GSS_ of DUT-AT7 and AT8 increase to the mA range, and I_DSS_ increases to the microampere (µA) range when failure occurs. However, DUT-AT9’s I_DSS_ remains unchanged.

**Figure 14:**
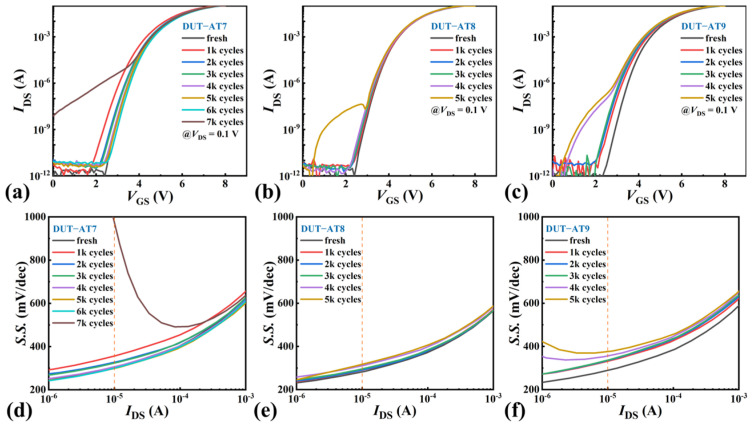
Degradation of the transfer curves and subthreshold swing for the DUTs during the repetitive surge current test of 60% SSC_max_: (**a**,**d**) DUT-AT7, (**b**,**e**) DUT-AT8, and (**c**,**f**) DUT-AT9.

**Figure 15:**
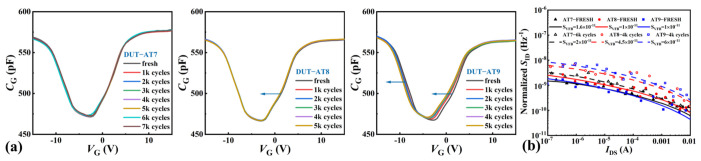
(**a**) Cg-Vg characteristic and (**b**) degradation of normalized noise power spectral density for DUT-AT7, AT8, and AT9.

In agreement with the degradation trend under large current repetitive surge stress, the V_TH_ degradation (Figure 13), transfer characteristics and S.S. (Figure 14), Cg-Vg characteristics, and LFN characteristics (Figure 15a,b) of the tested devices all exhibit a consistent trend. This indicates that more negative V_GS_ leads to more defect generation in the gate oxide layer, which results in more obvious V_TH_ degradation during the repetitive surge test at 60% SSC_max_.

## 4. Failure Mechanism Analysis

**Figure 16:**
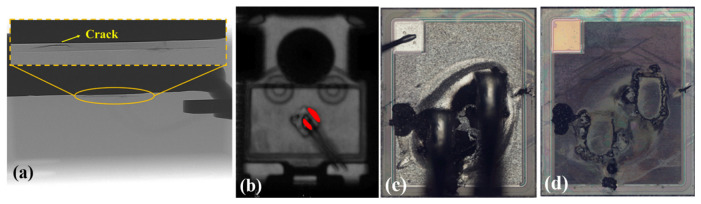
DUT-AT3: (**a**) X-ray inspection result. (**b**) SAM inspection result. (**c**) Surface morphology after decapsulation and before Al removal. (**d**) Surface morphology after decapsulation and Al removal.

**Figure 17:**
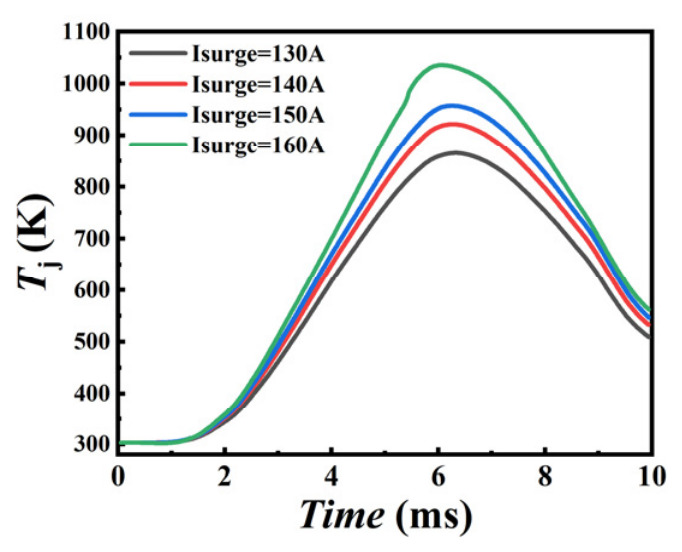
Estimated transient junction temperature of DUT-AT3 at different surge currents.

To study the failure mechanisms of AT SiC MOSFETs for single and repetitive surge current stress, failure analyses for DUT-AT3, AT6, and AT9 have been conducted, respectively. The X-ray inspection results for DUT-AT3 are shown in Figure 16a, where cracks are observed on the chip layer. Figure 16b displays the front-side SAM results, indicating delamination between the chip layer and the surrounding molding compound of DUT-AT3 after single surge stress. This is caused by the device temperature rising rapidly under single surge current stress, generating thermomechanical stress that causes structural changes in the overheated packaging material, corroborating the proposed failure physics in prior studies [31]. After decapsulation of the device, significant burn marks and cracks are observed near the source metal and around the gate bar, as shown in Figure 16c,d. The estimated junction temperatures are shown in Figure 17. For DUT-AT3, the localized junction temperature spiking is caused by the surge current of 160 A exceeding the melting point of Al (933 K), and the temperature rise accelerates the dielectric/interface degradation and leakage current increase. The abrupt increase in I_DSS_ and I_GSS_ near failure may then further enhance local power dissipation, forming positive electro-thermal feedback, consistent with the literature [15]. In contrast, when the surge current is below 140 A, the device junction temperature remains below the melting point of Al, which provides further evidence that repetitive surges induce cumulative degradation.

**Figure 18:**
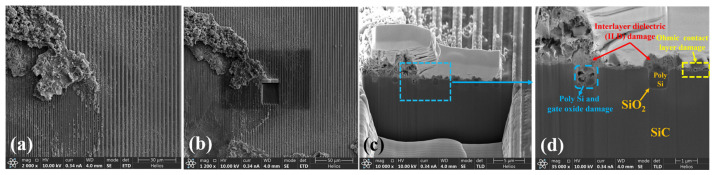
FIB-SEM analysis of failed DUT-AT3: (**a**,**b**) dye surface morphology and (**c**,**d**) a cross-section view of the damaged cell structure.

To further investigate the failure mechanism, focused ion beam (FIB) milling is performed on the area near the source metal burnout for DUT-AT3. As shown in Figure 18d, the melted source Al metal erodes and penetrates through the interlayer dielectric (ILD), which leads to the short circuit between the gate and the source. Meanwhile, the melted Al fills and breaks through the ohmic contact layer between the source metal and SiC doped region, which results in the degradation of the blocking capability. Both aforementioned factors ultimately lead to a three-terminal short circuit, which is consistent with the published literature [32,33].

**Figure 19:**
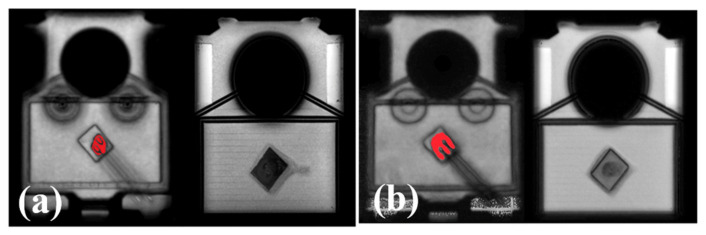
SAM inspection results of the DUTs: (**a**) DUT-AT6 and (**b**) DUT-AT9.

**Figure 20:**
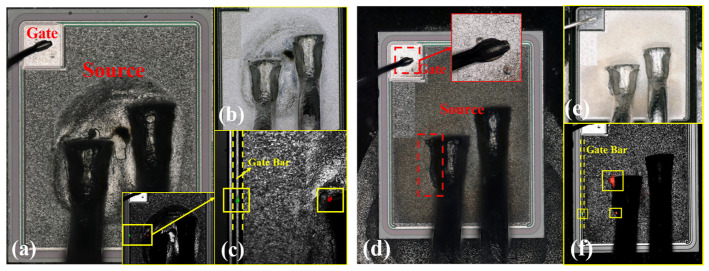
(**a**,**b**) Decapsulated chip surfaces and (**c**) OBIRCH localization of DUT-AT6. (**d**,**e**) Decapsulated chip surfaces and (**f**) OBIRCH localization of DUT-AT9.

**Figure 21:**
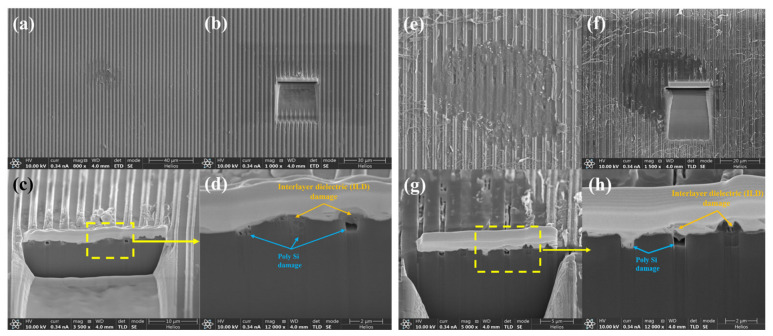
FIB-SEM analysis results: (**a**,**b**) dye surface morphology of DUT-AT6, (**c**,**d**) a cross-section view of the damaged cell structure of DUT-AT6, (**e**,**f**) dye surface morphology of DUT-AT9, and (**g**,**h**) a cross-section view of the damaged cell structure of DUT-AT9.

As shown in Figure 19, the SAM inspection results for DUT-AT6 and AT9 identify damage near the source bonding wires, accompanied by delamination of the encapsulant material (the red region). After the decapsulation of the devices, chip surface damage is observed by using a metallurgical microscope, as shown in Figure 19. Blackened carbonized marks are observed near the source bonding wires for DUT-AT6 and AT9, while no cracks are found on the chip surface. The severity of the burns for both DUT-AT6 and AT9 is lower than that of DUT-AT3, with the fewest burn marks for DUT-AT9. Further, leakage defect localization is performed by using the OBIRCH mode of emission microscopy (EMMI), as illustrated in Figure 20c,f. The red light indicates leakage locations, while the green light highlights impedance anomaly points. The green spot is located at the gate bar of the chip edge, while the red spot is located near the active region of the bond wires, and further FIB milling is conducted near the active region bond wire, followed by SEM scanning. As shown in Figure 21d,h, similar failure mechanisms have been observed for DUT-AT6 and AT9. The melted Al metal penetrates through the ILD and eventually leads to the short circuit between the gate and the source after repetitive surge current stress. Different from that of single surge current stress, the ohmic contact layer remains intact without obvious damage.

The device’s lifetime and failure mechanisms are intrinsically linked to its structural characteristics during the surge stress test. During third-quadrant surge operation, the current is shared by the intrinsic body diode and the MOS-channel-assisted conduction path. The asymmetric trench layout and the deep p+ shielding region are beneficial for reducing the on-state resistance and protecting the trench-bottom oxide under normal operation. However, during high surge current stress, these structural features also affect the internal current distribution. Since the body-diode path dominates (as shown in Figure 3c), the surge current mainly flows through deep p+ shielding region/n-drift junction system. Due to the asymmetric cell geometry, current crowding may occur near the trench sidewall in the deep p+ shielding corner [34]. Such nonuniform current distribution can result in localized Joule heating and enhanced electrothermal stress near the SiC/SiO_2_ interface, thereby accelerating interface-state generation, oxide-related degradation, and leakage current increase under repetitive surge stress.

Increasing the channel current contribution reduces the body-diode current component, lowers the effective V_SD,_ and decreases the surge energy dissipated in the bipolar diode path. Therefore, the localized heating and carrier-injection-induced degradation around the body diode and deep p+ shielding region can be mitigated. This means a larger channel current contribution at V_GS_ = 0 V is beneficial for improving repetitive surge reliability.

## 5. Conclusions

The reliability of asymmetric trench-gate SiC MOSFETs under single and repetitive surge current stresses (90% SSC_max_ and 60% SSC_max_) at different gate bias voltages (V_GS_ = 0 V, −5 V, and −10 V) has been systematically investigated.

When the device undergoes single surge current stress, it is indicated that V_GS_ does not affect the surge capability of the DUTs, with the same maximum surge current of 150 A. However, V_GS_ has influenced the degradation of key parameters. As V_GS_ becomes more negative, the degradation of V_TH_ becomes more obvious, with a decrease of up to 33.34% for AT3. This may be the result of a more negative V_GS_ leading to the increase in SiC/SiO_2_ interface defect density, intensifying gate-oxide deterioration, which is verified by the results of the transfer characteristics, S.S., Cg-Vg characteristics, and LFN characteristics. It is indicated from the microscopic analysis that the failure mode involves both chip failure and package failure. This is attributed to thermal runaway caused by a large single surge current, which leads to the melted source Al metal penetrating through the interlayer dielectric and the ohmic contact layer between the source metal and the SiC-doped region, respectively. It results in not only the short circuit between the gate and the source but also degradation in the blocking capability, ultimately leading to a three-terminal short circuit.

Different from a single surge current, it is indicated that V_GS_ has a significant impact on surge capability from the results of the large current (90% SSC_max_) repetitive surge stress. When V_GS_ = 0 V, the devices can withstand the maximum number of surge cycles with the least parameter degradation. The number of surge cycles the devices can withstand decreases as V_GS_ becomes more negative. This may be caused by a weaker contribution of the channel-assisted leakage conduction during repetitive current stress. As with the single surge stress, both chip and package failure occur for the large current (90% SSC_max_) repetitive surge stress. The device failure is caused by the melted Al metal penetrating through the interlayer dielectric and eventually leading to the short circuit between the gate and the source after repetitive surge current stress. Different from that of single surge current stress, the ohmic contact layer remains intact without obvious damage.

Compared with large surge current stress, the device can withstand several hundred times more surge cycles under small current (60% SSC_max_) repetitive surge stress. The maximum cycles are still observed at V_GS_ = 0 V, with a downward trend as V_GS_ becomes more negative. The failure mode and mechanism remain consistent with the large surge current repetitive tests.

To enhance the surge reliability of asymmetric trench-type SiC MOSFETs, it is recommended to increase the proportion of channel current in the total surge current, which reduces conduction losses and mitigates thermal runaway risks. Therefore, it is advised to operate such devices at V_GS_ = 0 V in the third quadrant.

## Figures and Tables

**Table 1:** Rated parameters and test conditions of the DUTs.

V_rated_ (V)	I_rated_ (A)	V_GS_ (V)	Surge Modes		
			Single-Pulse	Repetitive (90% SSCmax)	Repetitive (60% SSCmax)
1200	36	0	AT1	AT4	AT7
		−5	AT2	AT5	AT8
		−10	AT3	AT6	AT9

**Table 2:** Estimated parameters under different V_GS_ bias conditions.

V_GS_ (V)	Measured SSC_max_ (A)	V_SD_ at Same I_SD_ (V)	E_pluse_ (J)	Estimated T_j_ (K)
−10	150	7.14	4.75	949.7
−5		6.98	4.66	933.3
0		7.24	4.81	959.2

**Table 3:** Surge capability and three-terminal resistances when the DUTs fail.

DUTs	I_FSM_ (A)	Surge Capability	R_GS_ (Ω)	R_SG_ (Ω)	R_DG_ (Ω)	R_GD_ (Ω)	R_DS_ (Ω)	R_SD_ (Ω)
AT1	160	4.44	25	24	0.2	0.2	25.4	25.4
AT2			23.2	23.2	1.2 k	1.1 k	1.1 k	1.2 k
AT3			19	19	10	10	20	20

**Table 4:** The maximum surge cycles and three-terminal resistances when the devices fail (90% SSC_max_).

DUTs	I_surge_ (A)	Cycles	R_GS_ (Ω)	R_SG_ (Ω)	R_DG_ (Ω)	R_GD_ (Ω)	R_DS_ (Ω)	R_SD_ (Ω)
AT4	135	40	47.3	47.2	0.6 M	1.5 M	1.5 M	0.6 M
AT5		30	108.9	108.9	14.5 M	-	-	15.1 M
AT6		20	1.1 k	1.1 k	-	-	-	-

**Table 5:** The maximum surge cycles and three-terminal resistances when the devices fail (60% SSC_max_).

DUTs	I_surge_ (A)	Cycles	R_GS_ (Ω)	R_SG_ (Ω)	R_DG_ (Ω)	R_GD_ (Ω)	R_DS_ (Ω)	R_SD_ (Ω)
AT7	90	7500	3.1 k	3.1 k	20.9 M	-	-	21.1 M
AT8		6000	1.1 k	1.1 k	-	-	-	-
AT9		5500	0.7 k	0.7 k	-	-	-	-

## Data Availability

The data presented in this study are available upon request from the corresponding author.
